# Multi-comparative systems biology analysis reveals time-course biosignatures of *in vivo* bovine pathway responses to *B.melitensis*, *S.enterica* Typhimurium and *M.avium paratuberculosis*

**DOI:** 10.1186/1753-6561-5-S4-S6

**Published:** 2011-06-03

**Authors:** L Garry Adams, Sangeeta Khare, Sara D Lawhon, Carlos A Rossetti, Harris A Lewin, Mary S Lipton, Joshua E Turse, Dennis C Wylie, Yu Bai, Kenneth L Drake

**Affiliations:** 1 Department of Veterinary Pathobiology, College of Veterinary Medicine & Biomedical Sciences, Texas A&M University, College Station, TX 77843-4467, USA; 2 Instituto de Patobiologia, CICVyA-CNIA, INTA, CC25 (B1712WAA), Castelar, Buenos Aires, Argentina; 3 Department of Animal Sciences, University of Illinois at Urbana-Champaign, IL 61821, USA; 4 Fundamental and Computational Sciences Directorate, Biological Sciences Division, Macromolecular Structure and Dynamics, Pacific Northwest National Laboratory, Richland, WA 99352, USA; 5 Current address: Veterinary Microbiology & Pathology, College of Veterinary Medicine, Washington State University, P. O. Box 7040, Pullman, WA 99164, USA; 6 Seralogix LLC, 3839 Bee Cave Road, Austin, TX, 78746, USA

## Abstract

**Background:**

To decipher the complexity and improve the understanding of host-pathogen interactions, biologists must adopt new system level approaches in which the hierarchy of biological interactions and dynamics can be studied. This paper presents the application of systems biology for the cross-comparative analysis and interactome modeling of three different infectious agents, leading to the identification of novel, unique and common molecular host responses (biosignatures).

**Methods:**

A computational systems biology method was utilized to create interactome models of the host responses to *Brucella melitensis* (BMEL), *Salmonella enterica* Typhimurium (STM) and *Mycobacterium avium paratuberculosis* (MAP). A bovine ligated ileal loop biological model was employed to capture the host gene expression response at four time points post infection. New methods based on Dynamic Bayesian Network (DBN) machine learning were employed to conduct a systematic comparative analysis of pathway and Gene Ontology category perturbations.

**Results:**

A cross-comparative assessment of 219 pathways and 1620 gene ontology (GO) categories was performed on each pathogen-host condition. Both unique and common pathway and GO perturbations indicated remarkable temporal differences in pathogen-host response profiles. Highly discriminatory pathways were selected from each pathogen condition to create a common system level interactome model comprised of 622 genes. This model was trained with data from each pathogen condition to capture unique and common gene expression features and relationships leading to the identification of candidate host-pathogen points of interactions and discriminatory biosignatures.

**Conclusions:**

Our results provide deeper understanding of the overall complexity of host defensive and pathogen invasion processes as well as the identification of novel host-pathogen interactions. The application of advanced computational methods for developing interactome models based on DBN has proven to be instrumental in conducting multi-conditional cross-comparative analyses. Further, this approach generates a fully simulateable model with capabilities for predictive analysis as well as for diagnostic pattern recognition. The resulting biosignatures may represent future targets for identification of emerging pathogens as well as for development of antimicrobial drugs, immunotherapeutics, or vaccines for prevention and treatment of diseases caused by known, emerging/re-emerging infectious agents.

## Background

The complexity of host-pathogen interactions requires a system level understanding of the entire hierarchy of biological interactions and dynamics. A systems biology approach can provide systematic insights into the dynamic/temporal difference in gene regulation, interaction, and function, and thereby deliver an improved understanding and more comprehensive hypotheses of the underlying mechanisms [1,2]. Moreover, the ability to consolidate complex data and knowledge into plausible interactome models is essential to promote the effective discovery of key points of interaction. Accordingly, a systems biology approach to study molecular pathway gene expression profiles of host cellular responses to microbial pathogens holds great promise as a methodology to identify, model and predict the overall dynamics of the host-pathogen interactome.

## Methods

### Gene expression data acquisition

An established *in vivo* perinatal calf ligated ileal loop model, in conjunction with custom bovine microarrays, was used to study the early temporal changes in the host response to a previously optimized dosage of 1 x 10^9^ colony forming units of STM, BMEL, or MAP at four common sampling time-points post infection (0.5, 1, 2, and 4 hours pi) conducted under approved protocols by the Texas A&M University Institutional Animal Use and Care Committee. Gene expression data was collected by Dr. Adams’ lab at Texas A&M College of Veterinary Medicine following a surgical and sample collection methodology described elsewhere [3-6]. For each pathogen condition, under approved BSL2/BSL3 conditions, there were four biological replicate of infected and control loops in the non-survival surgeries for each pathogen performed on 3-week old male pathogen-free Holstein calves. Host RNA (from each host-pathogen surgery) was collected and co-hybridized in quadruplicate against bovine reference RNA to 13K custom bovine arrays (fabricated by W. M. Keck Center, University of Illinois at Urbana-Champaign) to allow for cross-comparison between experimental conditions. These custom microarrays consist of 70-mer oligonucleotides representing 13,258 unique oligos with 12,220 cattle ORFs. A detailed description of the design and development of the microarray has been published elsewhere [7]. Briefly, the microarray was comprised of unique 70-mers oligonucleotide sets representing cattle ORFs obtained from bovine non-immune related placenta and immune-related spleen cDNA libraries and based upon the earlier cDNA array platform GPL2864 and subtracted cDNA libraries created from embryonic and extra-embryonic tissues (NCBI libraries 15993, 15993 and 17188). Time matched RNA from non-infected ligated ileal loops were used as healthy state controls. Proteomics analysis of the STM-Host samples was provided by Pacific Northwest National Labs (PNNL) utilizing an approach referred to as the accurate mass and time (AMT) tag approach [8].

### Systems biology analysis and modeling

Computational analysis and modeling was completed by Dr. Drake at Seralogix using an integrated platform enabling a systems biology computational pipeline for multi-conditional analysis and modeling, termed the BioSignature Discovery System (BioSignatureDS^™^). Its core tools are based on Dynamic Bayesian Networks (DBN) [9], an advanced form of machine learning and pattern recognition. The platform enables comprehensive cross-comparisons of the genomic/proteomic data to identify key pathway/GO perturbations and underlying mechanistic regulatory points. BioSignatureDS^™^ is used to identify groups and individual genes that capture the perturbation in a pathway or biological process over time. This technique is named Dynamic Bayesian Gene Group Activation (DBGGA). DBGGA employs Dynamic Bayesian network (DBN) models that are trained using gene expression data replicates from the condition considered as controls. The other experimental condition expression data replicates are then used as evidence to test the goodness-of-fit of these data against the trained DBN control model. Goodness-of-fit is determined by Bayesian likelihood ratio tests that are subsequently transformed to a z-score test statistic (Bayesian z-score) to permit comparison of scores across all pathways and biological processes. The DBGGA computational method scores and rank groups of interrelated genes within a given pathway or gene ontology group across all time points in lieu of just one gene in a single time point (such as used in traditional t-tests) and thus determines the differences and commonalities between experimental and control conditions. DBGGA can also determine which genes are the significant sources of the perturbation. Such genes are designated as “candidate mechanistic genes”, a term we coined to describe those genes within a pathway that individually contributed significantly to the overall pathway Bayesian z-scores and thus are considered mechanistic candidates that may play key roles in governing the host response. Only those genes which are associated with a given pathway or biological process (GO group) were examined using the DBGGA modeling approach.

BioSignatureDS^™^ utilizes the significantly perturbed pathways, GO groups and the “mechanistic genes” as building blocks to construct system level network model of the disease/condition. Encapsulating global time-course patterns and multi-conditional behaviors of a large group of genes/proteins, the systems level model has great discriminating power even when the effects of individual genes are small. Thus, the disease models can be used for more efficient comparative modeling, pattern recognition (diagnostics) and simulations (prognostics). Further, proteomic data can be integrated into the models as an overlay to individual pathway and system-level models to both confirm the presence of proteins for their encoding genes as well as to visualize the temporal patterns of protein abundance.

## Results

For each host/pathogen interaction condition, BioSignatureDS^™^ modeled and scored 219 known metabolic and signaling pathways and 1620 biological processes (gene groups associated with Gene Ontology (GO) terms) at four time points. DBGGA was employed to identify the perturbations between pathogen conditions for pathways, GO categories, and genes. The DBGGA method generates Bayesian log likelihood scores that are normalized and transformed to a z-score equivalent (hereafter Bayesian z-score) so that all pathways and GO groups across all host/pathogen conditions can be equivalently compared and assessed for significance.

### DBGGA pathway analysis

**Figure 1:**
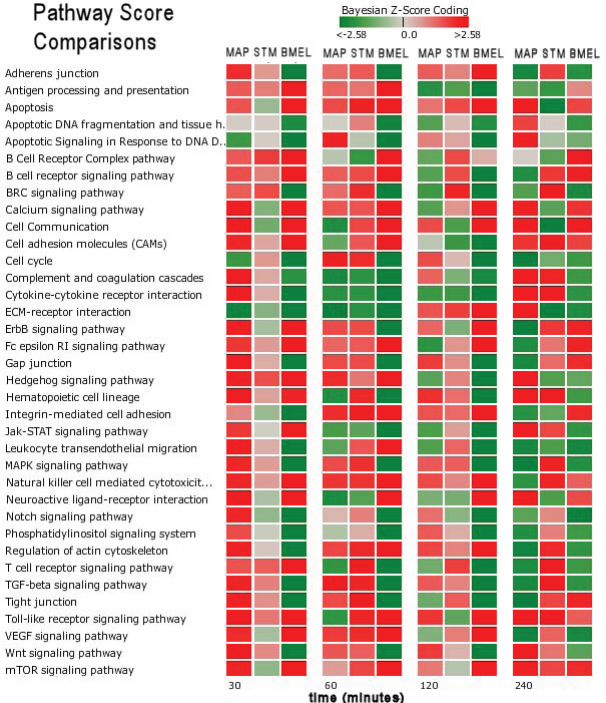
**Heat map comparison of pathway scores for each host condition by sampling time point post infection.** The score magnitudes are shown as a gradient color from light to bright red for induced and from light to bright green for suppressed pathway activity. *Brucella melitensis* (BMEL), *Salmonella enterica* Typhimurium (STM) and *Mycobacterium avium paratuberculosis* (MAP).

Of the 219 signaling/metabolic pathways scored, we focused on a subset of immune response related pathways as listed in Figure 1. This figure shows a heat map comparison of pathway Bayesian z-scores between pathogen conditions over time post infection. There were considerable differences between the host response profiles. MAP had strong early (30 minute) induction of the majority of its pathways and appeared to reverse to a more suppressive state by 240 minutes. STM’s early response indicated mild perturbations at 30 minutes that increased over time until several pathways were strongly induced by 240 minutes. BMEL was more strongly suppressive for the majority of pathways over time. At early times (30, 60 minutes) there were a few commonly induced pathways: Antigen Processing and Presentation, B Cell Receptor Signaling, Fc epsilon RI Signaling, Hedgehog Signaling, and Natural Killer Cell Mediated Cytotoxicity. In contrast, only ECM-receptor Interaction, Apoptotic Signaling and Apoptotic DNA Fragmentation had similar suppressions at 30 and 60 minutes. Interestingly, there was no single pathway at later times (120, 240 minutes) with similar perturbed states, implying that the host defenses have divergent biosignatures against the various virulent mechanisms presented by the pathogens.

**Figure 2:**
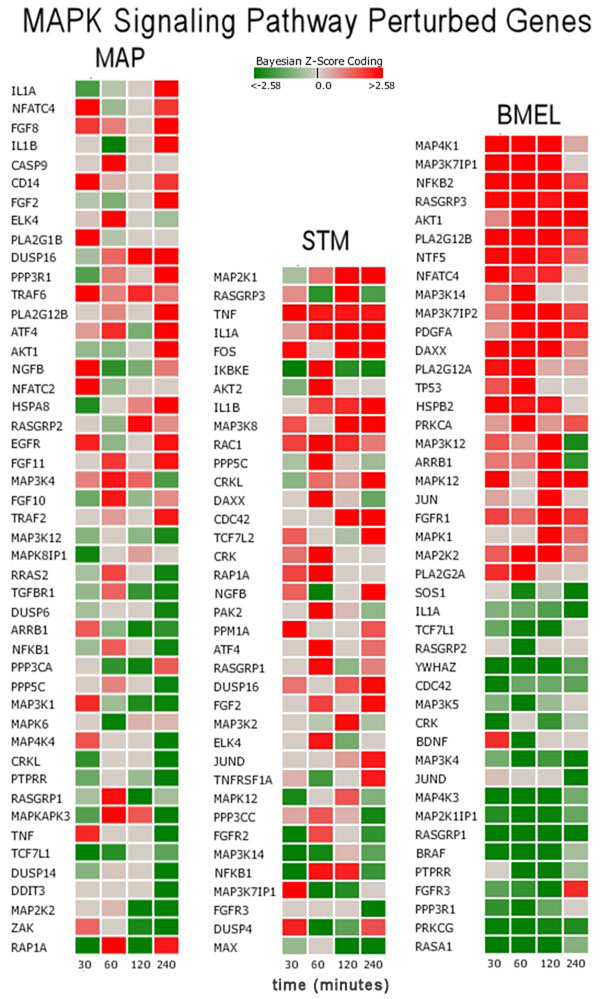
**Biosignature heat maps of gene scores for the MAPK signaling pathway for each host-pathogen condition***. Brucella melitensis* (BMEL), *Salmonella enterica* Typhimurium (STM) and *Mycobacterium avium paratuberculosis* (MAP).

Significant divergent responses between conditions were observed for MAPK Signaling. For MAP, the MAPK pathway reversed from induced to suppressed, while STM increased induction and BMEL maintained a suppressed state. The MAPK Signaling Pathway was implicated in bacterial pathogenesis for a number of pathogens such as *Salmonella enterica* serovar Typhimurium [10], *Yersinia* spp*.*[11], *Listeriamonocytogenes*[12], and *Mycobacterium* spp. [13]. This pathway was selected for more detailed discussion with regard to gene perturbations and mechanistic interpretations. Figure 2 is a heat map of significantly perturbed genes for the MAPK pathway by pathogen condition. In this figure, the genes are sorted in order of highest up modulation to lowest down modulation, and for a gene to be included in this figure, a Bayesian z-score>|2.24| at any one time point was required. The Bayesian z-score > |2.24| reflects 99% confidence in the data. It is easy to observe that the perturbed genes and their expression patterns are quite different between conditions. Surprisingly, of the 171 measured genes on this pathway, only two genes in Figure 2 were found to be commonly perturbed across all three pathogen conditions: 1) *IL1A*, which encodes interleukin 1 protein involved in various immune responses, inflammatory processes, and hematopoiesis; and 2) *RASGRP1*, which encodes a protein characterized by the presence of a Ras superfamily guanine nucleotide exchange factor (GEF) domain that activates the Erk/MAP kinase cascade and regulates T-cell and B-cell development, homeostasis and differentiation. The perturbation of *IL1A* and *RASGRP1* is consistent with genes involved in immune response, but the expression patterns for these two genes vary significantly between pathogens.

**Figure 3:**
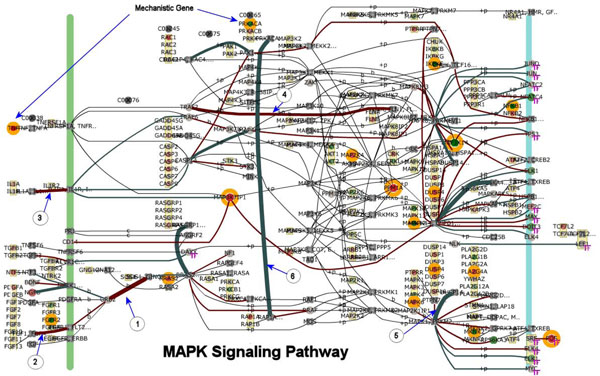
**MAPK pathway network model for bovine host infected with *Salmonella enterica* Typhimurium (STM)**. The model is trained and then used to score the pathway and individual genes. This figure is the MAPK pathway as a screen capture taken from BioSignatureDS™ user interface. This figure is a snapshot of the perturbed state of the MAPK pathway at 60 minutes post infection for the STM-Host condition. Mechanistic genes are encircled with an orange ring. In the pathway, genes aligned along the green vertical bar are typically receptor/membrane related, and those on the blue bar are nucleus related. Gene nodes with an attached “TF” subscript are transcription factors or transcription factor related. The arcs connecting genes are coded to indicate the correlation between connected genes. Brown arcs indicate positive correlation while turquoise arcs represent negative correlations, and the thickness of the arc indicating the magnitude of correlation. Arcs with an encircled number and arrow correspond to those arcs labeled accordingly in Table 1.

Simply comparing and contrasting the expression patterns of perturbed genes was inadequate for deciphering the MAPK pathway response dynamics. Clearly, the uniqueness of the MAPK pathway responses suggested that very different invasion/evasion mechanisms have evolved for each pathogen. More sophisticated methods are needed to identify potential points of host response disruptions. This is done by interrogating the trained DBN model for the MAPK Pathway for genes that exceed threshold Bayesian z-scores>|2.24| (“mechanistic genes”) and gene-gene network relationships (arcs). For example, Figure 3 shows the visualization of the MAPK pathway network. The network can be employed to visualize several key features that would otherwise be difficult to discern by looking at spread sheet lists of genes. For example, the state of gene modulation is distinguished by color coded nodes. The state of upstream and downstream genes can be easily identified. Various threshold levels can be entered to identify significantly perturbed genes (annotated with orange circles, Fig 3). The strength of correlation between gene pairs is indicated by the color and thickness of the arcs connecting the genes.

**Table 1:** Arc weight correlation

Arc Label	Gene Start	Normalized Weight	Gene End	Relation Type
**1**	SOS1	**0.372**	GRB2	binding
**2**	FGF1	**0.306**	FGFR2	activation
**3**	IL1B	**0.271**	IL1R2	activation
**4**	TRAF2	**0.241**	FLNA	binding
	FGF1	**0.234**	FGFR4	activation
	MAPK4	**0.204**	PLA2G12B	activation
	MAPK8IP1	**0.202**	MAP4K1	binding
	DUSP4	**0.188**	MAPK3	inhibition
	MAPK4	**0.185**	MAP2K1IP1	binding
	DUSP4	**0.178**	MAPK6	inhibition
	MAPK6	**0.176**	RPS6KA3	phosphorylation
	MAPK12	**0.173**	TP53	phosphorylation
	MAP3K7IP1	**0.092**	MAPK12	phosphorylation
	CASP9	***-0.008***	PAK1	phosphorylation
	CASP7	***-0.123***	MAP4K1	phosphorylation
	RASGRP4	***-0.175***	RRAS2	activation
	MAPK12	***-0.184***	RPS6KA5	phosphorylation
	MAPK1	***-0.191***	YWHAZ	activation
**5**	DUSP4	***-0.199***	MAPK4	inhibition
**6**	PRKACB	***-0.209***	RAP1B	activation

Table shows highly correlated (positive weights) or anti-correlated gene-gene relationships (negative weights) learnt from the model training. The arc label column with numbers 1-6 correspond to the arcs labeled in Figure 3.

In Table 1, we show a list of 20 specific gene-to-gene relations associated with the MAPK pathway (Figure 3) having strong positive and/or negative arc weight correlations. We normalized the DBN arc weights to allow equivalent comparison to other pathway gene-gene relations. In this arc weight table, a few significant relationships are labeled (1 thru ) in the table and on the network (Figure 3) with an encircled number and arrow pointing to the corresponding arc. It is hypothesized that virulence factors from each pathogen can have different disruptive influences on the host’s MAPK Signaling Pathway and that such disruption can be used to identify pathogenic mechanisms unique to each pathogen. For example, the relation arc *TRAF2*->*FLNA* had a strong positive weight (correlated) for STM-Host (0.241) and for the BMEL-Host (0.204) while MAP-Host had a large negative weight (anti-correlated) of -0.17. The reversal of gene-to-gene arc weight of MAP-Host may indicate a disruption of either *TRAF* or *FLNA* gene by virulent factors of the pathogens, identifying potential novel points of interaction. *TRAF2* encodes a protein that is a member of the TNF receptor associated factor (TRAF) protein family. This protein is required for TNF-alpha-mediated activation of MAPK8/JNK and NF-κB. It has a binding relationship with the filamin-A protein encoded by the *FLNA* gene. *FLNA* participates in the anchoring of membrane proteins to the actin cytoskeleton. This type of interaction analysis can be done for every pathway and used to identify novel differences between pathogen conditions. The visualization of mechanistic genes and arc weight enables an efficient identification of the differences between pathogenic influences.

### DBGGA gene ontology analysis

**Figure 4:**
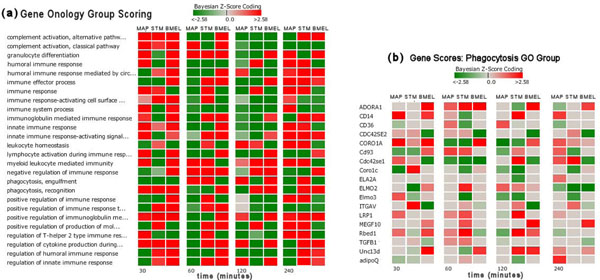
(a) Gene ontology group scores using DBGGA A few groups are displayed here to illustrate the GO group scoring difference between pathogen conditions. (b) Gene scores for phagocytosis GO group Illustrating the temporal difference of gene expression Bayesian z-score for each pathogen condition.

A DBGGA analysis was also conducted for gene GO categories. For each pathogen condition, 1620 biological process GO categories were scored. Each condition produced its own unique set of highly scored GO functions, but for comparison purposes, we chose a small subset of highly perturbed categories to illustrate the different temporal responses as shown in Figure 4(a). Figure 4(b) illustrates the comparative analysis of gene scores for the phagocytosis GO Group.

### Biological system model generation for comparative host response analysis

Utilizing BioSignatureDS^™^, the significantly perturbed pathways and gene groups from DBGGA were integrated to construct a plausible system level model of the disease/condition. The system model encompasses whole time-course patterns and multi-conditional behaviors of a large group of genes/proteins. The system model can be used for more efficient comparative modeling, pattern recognition and simulation supporting “what-if” type of analyses. The system model is constructed from a method based on merging of pathways with known gene/protein relationships and produces a trained and optimized network model similar to the pathway network shown in Figure 3. Following this procedure, a system model was constructed from 13 selected pathways (listed in Table 2) showing significant perturbation across the three pathogen conditions. As can be seen in this table, pathways have a remarkable difference in perturbation that is exploited for identifying unique and common gene patterns. The resulting model has a common network structure that is trained using the host response data. This model is employed for comparing biosignatures between the different pathogen host responses and was comprised of 622 genes and over 750 gene-to-gene relations. We trained three models, each with data from a different host-pathogen condition. For each model and each host-pathogen time point data sample, we tested the hypothesis that the sample did not fit the model at a confidence level of 95%. Table 3 provides the results of this testing in terms of sensitivity and specificity for each of the models listed.

**Table 2:** Pathways Utilized for System Model Generation

KEGG ID	Pathway Description	Category	STM	BMEL	MAP
hsa04810	Regulation of actin cytoskeleton	Cell Motility	2.13	3.66	-2.45
hsa04664	Fc epsilon RI signaling	Immune System	1.49	3.8	-2.12
hsa04660	T cell receptor signaling	Immune System	2.58	-1.8	-2.14
hsa04630	Jak-STAT signaling pathway	Signal Transduction	1.72	-1.9	3.2
hsa04620	Toll-like receptor signaling	Immune System	2.75	2.92	2.8
hsa04540	Gap junction	Cell Communication	1.65	2.06	2.6
hsa04512	ECM-receptor interaction	Signaling Molecules/Interaction	-2.86	3.2	2.64
hsa04370	VEGF signaling pathway	Signal Transduction	2.06	4.44	-2.39
hsa04340	Hedgehog signaling pathway	Signal Transduction	1.61	-1.34	2.61
hsa04310	Wnt signaling pathway	Signal Transduction	1.38	-1.49	3.01
hsa04210	Apoptosis	Cell Growth and Death	-2.65	3.72	3.04
hsa04150	mTOR signaling pathway	Signal Transduction	1.71	5.21	2.33
hsa04010	MAPK signaling pathway	Signal Transduction	2.28	-2.07	-2.84

Values shown are the Bayesian z-score for each pathway, within a given organism.

**Table 3:** Sensitivity and Specificity Model Assessment

True Model	Time Pt	False Pos Rate	Specificity	False Neg Rate	Sensitivity
BMEL-Host	30	0.00	1.00	0.34	0.66
BMEL-Host	60	0.00	1.00	0.30	0.70
BMEL-Host	120	0.00	1.00	0.32	0.68
BMEL-Host	240	0.25	0.75	0.25	0.75
BMEL-Host	All	0.06	0.94	0.30	0.70
STM-Host	30	0.00	1.00	0.50	0.50
STM-Host	60	0.00	1.00	0.36	0.64
STM-Host	120	0.00	1.00	0.50	0.50
STM-Host	240	0.25	0.75	0.32	0.68
STM-Host	All	0.06	0.94	0.42	0.58
MAP-Host	30	0.00	1.00	0.50	0.50
MAP-Host	60	0.00	1.00	0.57	0.43
MAP-Host	120	0.00	1.00	0.52	0.48
MAP-Host	240	0.25	0.75	0.52	0.48
MAP-Host	All	0.06	0.94	0.53	0.47

### Cross-species protein-protein interactome model

Ultimately, the goal is to develop interactome models between the pathogen and the host to make predictions of protein-protein interactions (PPIs). A preliminary STM-Host model (model not shown herein) was created using BioSignatureDS™ augmented with new computational methods to learn PPIs between the pathogen and the host. Model PPI structure learning utilized (host and STM) microarray gene expressions and mass spec protein levels (extracted and measured simultaneously from the bovine host [14,15]) guided by *a prior* biological interactions and cross-species PPI predictions. The model identified a set of 34 known and novel STM-Host protein interactions. These predicted interactions will be employed in guiding future biological experiments for their confirmation as host-pathogen virulence factors and are candidates for points of intervention. PPI interactome models will be developed for BMEL-Host and MAP-Host and full results reported in future publications.

## Conclusions

We analyzed the set of system model genes (622) looking for strong differential expressions (99% confidence) between the three conditions (STM-Host vs. MAP-Host, STM-Host vs. BMEL-Host, MAP-Host vs. BMEL-Host). In this manner we were able to identify sets of genes that were uniquely different from the other two pathogen responses. We found 69 unique genes in the BMEL-Host, 253 genes in the STM-Host, and 64 genes in the MAP-host. Listing of these genes is not provided herein, but will be made publically available in a forthcoming publication. The results of our analysis and modeling identified common and uniquely perturbed genes and biosignatures. A cross-species interactome model identified 34 PPIs that was learned from STM-Host co-expressed gene and protein data. More importantly, these models produce gene networks that can be used to identify novel targets for intervention, as well as the overall improved understanding of the underlying pathogenicity of each pathogen.

## Competing interests

Dr. Drake, Dr. Wylie, and Dr. Bai participated in the analysis and modeling presented in this paper. They all work for Seralogix, a private business that is developing the analysis and modeling software for commercial purposes.
